# Phase II Study of Biweekly Plitidepsin as Second-Line Therapy for Advanced or Metastatic Transitional Cell Carcinoma of the Urothelium

**DOI:** 10.3390/md7030451

**Published:** 2009-09-16

**Authors:** Herlinde Dumez, Enrique Gallardo, Stephane Culine, Joan Carles Galceran, Patrick Schöffski, Jean P. Droz, Sonia Extremera, Sergio Szyldergemajn, Aude Fléchon

**Affiliations:** 1 University Hospitals Gasthuisberg, Catholic University Leuven, Herestraat 49, 3000 Leuven, Belgium; E-Mail:patrick.schoffski@uz.kuleuven.ac.be (P.S.); 2 Corporació Parc Taulí, C/Parc Taulí, s/n, 08208 Sabadell (Barcelona), Spain; E-Mail:egallardo@tauli.cat (E.G.); 3 Parc Euromedicine, 323 rue des Apothicaires, 34298 Montpellier, France; E-Mail:stephane.culine@hmn.aphp.fr (S.C.); 4 Hospital Universitari del Mar, Passeig Marítim, 25-29, 08003 Barcelona, Spain; E-Mail:jocarles@vhebron.net (J.C.G.); 5 Centre Léon Bérard, 28 Rue Laennec, F-69008, Lyon, France; E-Mail:droz@lyon.fnclcc.fr (J.P.D.);flechon@lyon.fnclcc.fr (A.F.); 6 Pharma Mar S.A., Av. de los Reyes, 1, PI La Mina-Norte, 28770 Colmenar Viejo, Madrid, Spain; E-Mails:sextremera@pharmamar.com (S.E.);sszyldergemajn@pharmamar.com (S.S.)

**Keywords:** plitidepsin, TCC, urothelium, second-line, transitional cell carcinoma

## Abstract

The objective of this exploratory, open-label, single-arm, phase II clinical trial was to evaluate plitidepsin (5 mg/m^2^) administered as a 3-hour continuous intravenous infusion every two weeks to patients with locally advanced/metastatic transitional cell carcinoma of the urothelium who relapsed/progressed after first-line chemotherapy. Treatment cycles were repeated for up to 12 cycles or until disease progression, unacceptable toxicity, patient refusal or treatment delay for >2 weeks. The primary efficacy endpoint was objective response rate according to RECIST. Secondary endpoints were the rate of SD lasting ≥ 6 months and time-to-event variables. Toxicity was assessed using NCI-CTC v. 3.0. Twenty-one patients received 57 treatment cycles. No objective tumor responses occurred. SD lasting <6 months was observed in two of 18 evaluable patients. With a median follow-up of 4.6 months, the median PFR and the median OS were 1.4 months and 2.3 months, respectively. The most common AEs were mild to moderate nausea, fatigue, myalgia and anorexia. Anemia, lymphopenia, and increases in transaminases, alkaline phosphatase and creatinine were the most frequent laboratory abnormalities. No severe neutropenia occurred. Treatment was feasible and generally well tolerated in this patient population; however the lack of antitumor activity precludes further studies of plitidepsin in this setting.

## 1. Introduction

Transitional cell carcinoma (TCC) of the bladder affects 356,000 patients per year worldwide, with some 145,000 deaths resulting from the disease [1]. The standard first-line treatment for patients with advanced TCC is platinum-based combination chemotherapy like methotrexate, vinblastine, doxorubicin and cisplatin (MVAC), or gemcitabine and cisplatin (GC) [2–4]. Both combinations achieve high response rates, although MVAC is associated with significant toxicities and a toxic death rate of 3–4% [2,3]. Despite this, prognosis remains poor, with an expected median survival of approximately one year [5,6]. Single agent and second-line treatments with ifosfamide, gemcitabine, paclitaxel, docetaxel or pemetrexed [7–9] after prior MVAC, produce response rates of 11–28%, but OS do not seem to be improved and the drug-related toxicity remains an issue in this very frail patient population. Thus, there is a need for second-line regimens that either improve survival outcome or provide a similar one with reduced toxicity.

Plitidepsin is a cyclic depsipeptide originally isolated from the Mediterranean tunicate *Aplidium albicans*, currently produced by chemical synthesis. Plitidepsin is a COMPARE negative compound, whose main mechanism of action remains to be fully elucidated, although evidence suggests that oxidative stress [10], and decreased intracellular levels of glutathione ultimately lead to both caspases-dependant and independent cellular apoptosis induction [11], whereas activation of the Rac1-JNK pathway plays a major role [12,13]. In addition, plitidepsin exhibits antiangiogenic activity through the inhibition of the expression of genes like the vascular endothelial growth factor (VEGF) and its receptor (VEGFR-1) [14–17]. Plitidepsin was found to be active, *in vitro* and *in vivo*, usually at concentrations as low as in the nanomolar range [18]. Human bladder 5637 cell line was found to be particularly sensitive to plitidepsin, with a mean IC_50_ of only 3.3 × 10^−8^ M [19]. *In vivo*, plitidepsin also showed a strong antitumor activity against subcutaneously implanted HTB-9 human bladder cell lines in a murine model [20,21].

Two schedules were selected for further phase II clinical development: a weekly regimen of 3.2 mg/m^2^ on days 1, 8 and 15 every 4 weeks over 1-hour and the fortnightly regimen of 5.0 mg/m^2^ over 3-hours [19,20,22–25]. Evidence for objective remission and tumor control was found in patients with several advanced solid tumors and hematological malignancies [26]. The dose-limiting toxicities (DLTs) included myalgia, muscular weakness, transient and reversible transaminase increase and fatigue. Remarkably, plitidepsin lacks of clinically significant bone marrow toxicity.

The objective of this exploratory multicenter, open-label, single-arm, phase II clinical trial was to assess the antitumor activity and safety profile of plitidepsin 5 mg/m^2^ given every two weeks as a 3-hour intravenous (i.v.) infusion to patients with advanced TCC of the urothelium who relapsed or progressed after first-line chemotherapy.

## 2. Patients and Methods

Twenty-one patients participated in this study between October 2004 and December 2006 at five European medical centers. The protocol was approved by the institutional review board of each participating center, and a signed written informed consent was obtained from each patient before registration.

### 2.1. Patient population

The patients had unresectable, locally advanced or metastatic, and histologically-confirmed TCC of the bladder, ureter or renal pelvis, with documented progressive disease (PD) prior to registration and measurable disease according to the Response Evaluation Criteria In Solid Tumors (RECIST) [27]. All patients had to receive no more than one previous line of systemic chemotherapy for advanced disease. Prior radiotherapy was allowed, but a minimum of four weeks had to elapse between the end of the prior radiotherapy and patient enrolment. Patients were ≥ 18 years old, had an Eastern Cooperative Oncology Group (ECOG) performance status (PS) score of ≤ 2 and had recovered from any toxicity derived from previous treatments. Patients had an adequate organ function as defined by neutrophil count ≥ 1.5 × 10^9^ /L, platelet count ≥ 100 × 10^9^ /L, hemoglobin ≥ 9 g/dL, creatinine clearance ≥ 40 mL/min, serum bilirubin ≤1.5 mg/dL, alkaline phosphatase (AP) ≤ 2.5 × the institutional upper limit of normal (ULN) and up to ≤ 5 × ULN in case of extensive bone metastases, aspartate aminotransferase (AST) and alanine aminotransferase (ALT) ≤2.5 × ULN and up to ≤ 5 × ULN in case of liver metastases, albumin ≥25 g/L and normal left ventricular ejection fraction (LVEF).

Patients were excluded if they were pregnant or lactating women or were not using adequate contraception methods, had a history of other neoplastic diseases (with the exception of adequately treated non-melanoma skin carcinoma or carcinoma *in situ*), had known brain or leptomeningeal involvement, suffered from any other relevant medical condition that may harm study participation, or had known hypersensitivity to plitidepsin or to any component of the reconstitution solution (cremophor EL, mannitol and ethanol).

### 2.2. Study treatment

Plitidepsin (Pharma Mar, Colmenar Viejo, Madrid, Spain) was infused at a dose of 5 mg/m^2^ as a 3-hour i.v. infusion every 2 weeks (i.e., one cycle = two weeks). Treatment cycles were repeated for a maximum of 12 (~six months) or until disease progression, unacceptable toxicity, patient refusal or treatment delay for >2 weeks. In the event of specific toxicities after the first cycle, the dose of plitidepsin was to be reduced in subsequent cycles, first to 4.25 mg/m^2^ and then to 3.6 mg/m^2^. No further dose reductions were allowed.

Treatment with hematopoietic growth colony stimulating factors was allowed (except during the first cycle) in accordance with the guidelines of the American Society of Clinical Oncology (ASCO) [28]. Prophylactic treatment for emesis, which included glucocorticoids (dexamethasone 4–8 mg i.v.), H1-receptor and serotonin (5-HT_3_) receptor antagonists [29], was administered 20–30 min before each plitidepsin infusion. Patients developing grade ≥2 muscular toxicity could be empirically treated with L-carnitine (1.5 g every 8 hours) until returning to grade ≤1 severity.

### 2.3. Evaluations during the study

Radiological evaluation (chest X-ray, computer tomography [CT] scan or magnetic resonance imaging [MRI]) was required within four weeks before start of study treatment, and within six months prior to registration in order to document pre-baseline disease progression. While on treatment, a complete clinical examination was done at the end of each cycle (*i.e.*, every two weeks). Blood samples for hematological and biochemistry analyses were obtained weekly during the first eight weeks of therapy and afterwards at least every two weeks. Creatinine clearance calculation and urine analysis were assessed before every cycle administration, while coagulation tests were performed every other cycle. LVEF measurements were repeated at 3–6 months after the start of treatment. All disease parameters were reassessed every eight weeks by using appropriate imaging procedures for the assessment of measurable and non-measurable lesions as per RECIST.

### 2.4. Study endpoints

The primary efficacy endpoint was objective tumor response rate according to RECIST in all patients who received a minimum of two treatment cycles. Whenever the criteria of response were met, re-assessment had to be repeated at least four weeks later in order to confirm response. Any eligible patients who died or stopped treatment because of unmanageable toxicity prior to response evaluation were considered non-evaluable for response.

Secondary efficacy endpoints were the rate of stable disease (SD) lasting for at least six months and time-to-event variables (tumor response duration, time to progression [TTP], progression-free survival [PFS] and OS), and evaluation of the safety profile of plitidepsin. Patients were evaluable for toxicity if they had received at least one plitidepsin infusion. Treatment-related toxicities were graded according to National Institute of Health Common Toxicity Criteria (NCI-CTC), version 3.0 [30]. If toxicity persisted after the end of treatment, further assessments were performed 30 days after the administration of the last protocol treatment or at the resolution of all abnormalities that occurred during treatment.

### 2.5. Statistical analyses

A Simon’s two-stage MiniMax design was used to test the null hypothesis that the probability of objective response (confirmed PR or CR) was H0 ≤5% versus the alternative hypothesis that it was HA ≥ 20% [31]. After testing the drug on 18 patients in a first stage, the trial was to be terminated if no responses were observed. Otherwise, the trial was to go onto a second stage and other 14 patients were to be evaluated to reach a total of 32 patients evaluable for efficacy. If the total number of patients with an objective tumor response was ≤ 3, this schedule was not to be considered for further evaluation. Descriptive statistics were used to characterize demographics, response and toxicity rates and laboratory observations. Time-to-event efficacy endpoints and their fixed time point rates were analyzed according to the Kaplan-Meier method [32].

## 3. Results

### 3.1. Patient characteristics

Patient and disease characteristics at baseline are shown in Table 1. The median age was 64 years, 76.2% were males and 95.2% had an ECOG PS score of 0 or 1.

All patients had metastatic and/or locally advanced TCC, except for one who had poorly differentiated urothelial carcinoma. Patients had a median of one metastatic site (range, 1–3), with lymph nodes as the most common tumor site involved at baseline (52.4% of patients), followed by the liver (28.6%) and the lung (14.3%).

All patients had previously received chemotherapy (Table 2); additionally, three patients also received biological therapy (BCG vaccine two patients and trastuzumab one patient). Most patients (76.2%) had previously received cisplatin (81.0%) and gemcitabine (71.4%). All patients had undergone surgery, mostly consisting of radical cystectomy (71.4%), while radiotherapy had been administrated to 5 patients (23.8%) in the palliative setting.

### 3.2. Extent of exposure

A total of 57 cycles (median: two per patient; range: 1–8) were administered during the study. The median total cumulative dose delivered was 10.0 mg/m^2^ (range, 5.0–40.0 mg/m^2^) and the median dose intensity was 2.5 mg/m^2^/wk (range, 1.3–2.5 mg/ m^2^/wk), which corresponded to a median relative dose intensity of 99.4% (range, 52.1–101.0%).

Cycle delays occurred in four patients (19.0%); all due to reasons unrelated to plitidepsin. The length of delay was 3–7 days. Thirteen patients (61.9%) discontinued treatment early because of disease progression, while other patients discontinued due to death as a result of disease progression (n = 3, 14.3%), treatment refusal (n = 2, 9.5%) and other reasons (n = 2, 9.5%) such as general condition deterioration and worsening of Parkinson’s disease. Only one patient (4.8%) discontinued due to plitidepsin-related increase in creatine phosphokinase (CPK) levels after three cycles.

### 3.3. Assessment of efficacy

Three of 21 treated patients were non-evaluable for efficacy due to not having undergone any tumor assessments before discontinuing the study as a result of patient refusal (n = 2) and worsening of Parkinson’s disease (n = 1). No objective tumor responses were found among the 18 evaluable patients. Two patients (11.1%) achieved SD according to RECIST that lasted less than six months (2.2 months and 3.7 months). With a median follow-up of 4.6 months, the median PFS was 1.4 months (95% CI, 0.9–1.6 months) and the median OS was 2.3 months (95% CI, 1.4–4.6 months).

### 3.4. Toxicity

All 21 treated patients were assessable for toxicity. Nausea (38.1% of patients), fatigue (28.6%), myalgia (28.6%) and anorexia (23.8%) were the most frequent adverse events (AEs) related to plitidepsin. No plitidepsin-related grade four AEs were observed. No plitidepsin-related hospitalizations or deaths occurred during the study. Table 3 shows all the AEs reported during this study regardless of relationship observed in at least 5% of patients. One patient received only one administration of L-carnitine due to muscular weakness and increased CPK levels.

The most frequent biochemical abnormalities were transient and asymptomatic increase in AP (76.2% of patients), creatinine (61.9%) and hepatic transaminases (ALT: 61.9% and AST: 52.4%) (Table 4). Grade 3 AP increases occurred in 4 patients, whereas one patient had grade 3 ALT increase. Additionally, one patient had grade 3 bilirubin increase after one cycle. No patients had grade 3/4 increases in AST, creatinine or CPK levels.

The most common hematological abnormalities were anemia and lymphopenia, which were observed in 21 (100.0%) and 11 (55.0%) patients, respectively (Table 4). Anemia and lymphopenia were grade 3 in one patient each; however grade 3 lymphopenia was already present at baseline. Additionally, one patient had grade 4 lymphopenia after cycle 3. No patients showed leukopenia of any grade, whereas the cases of neutropenia and thrombocytopenia found during the study were grade 1 only.

## 4. Discussion

This exploratory, phase II study of plitidepsin 5 mg/m^2^ given every two weeks as a 3-hour infusion showed poor antitumor activity among 18 evaluable pretreated patients with locally advanced or metastatic TCC of the urothelium who relapsed or progressed after first-line chemotherapy. No objective tumor responses were found and only two patients achieved SD according to RECIST, but these were short-lasting (less than six months).

The optimal agents and regimens for second-line chemotherapy in advanced TCC still remain undefined. Both single-agent and combination regimens have been evaluated as potential therapies [33]. Recently, Di Lorenzo *et al.* have reported that the combination of paclitaxel and cyclophosphamide was well tolerated and associated with promising 31% response rate in previously treated patients [34]. With respect to single-agent chemotherapy, gemcitabine alone has shown a higher response rate (23–25%) in prior unexposed patients than docetaxel, paclitaxel and ifosfamide (10–20%) [7,35–38], although its incorporation as part of standard fist line regimens makes unclear its utility in the relapse setting. Unfortunately, among several newer compounds evaluated in this setting bortezomib [39], sunitinib [40], ixabepilone [41] and aflibercept [42] all have shown very limited or no efficacy. Only vinflunine, a novel vinka alkaloid class compound, has shown comparable activity to taxanes or ifosfamide in a large phase II study with a 14.6% response rate evaluated by the independent review committee (IRC) [43]. Thus, clearly newer active agents are needed.

In the current study, as a result of lack of objective responses with this biweekly plitidepsin regimen, patient recruitment did not progress into the second stage and the trial was early closed. Patient population was comparable to other studies regarding number of previous lines, disease extension and ECOG PS. Clinical benefit with single-agent plitidepsin has been found in patients with other advanced solid tumor types, particularly in chemo-refractory tumors as renal, melanoma, hepatocellular carcinoma and carcinoid tumors and in hematological malignancies (e.g., multiple myeloma and non-cutaneous peripheral T-cell lymphoma) [24].

Toxicity with plitidepsin in patients with locally advanced or metastatic TCC mostly consisted of mild or moderate AEs (nausea, fatigue, myalgia and anorexia) and was in accordance with the toxicities observed when plitidepsin was administered to patients with other malignancies [23,25]. The most common laboratory abnormalities were anemia and lymphopenia, and reversible and asymptomatic increases in transaminases, AP and creatinine. Neither clinically significant neutropenia nor thrombocytopenia occurred in this frail patient population.

## 5. Conclusions

In conclusion, the lack of significant antitumor activity despite a favorable safety profile suggests that further studies of plitidepsin as a single agent in TCC of the urothelium are not warranted; however its lack of overlapping toxicity with other chemotherapeutic agents, as well as the presence of additivity or synergy in preclinical models with compounds like carboplatin, gemcitabine and/or taxanes, open some new options for this drug in the future.

## Figures and Tables

**Table 1:** Patient and disease characteristics.

		No. of patients (n)*	%

**Gender**	Male	16	76.2
	Female	5	23.8

**Age (years)**	Median (range)	64 (41–72)	

**Performance status (ECOG)**	0	4	19.0
	1	16	76.2
	2	1	4.8

**Tumor extension at baseline**	Metastatic	14	66.7
	Locally advanced/metastatic	5	23.8
	Locally advanced	2	9.5

**Bajorin criteria**	Good risk	8	38.1
	Intermediate risk	13	61.9

**Number of sites involved**	Median (range)	1 (1–3)	

**Sites of disease at baseline**	Lymph node	11	52.4
	Liver	6	28.6
	Lung	3	14.3
	Soft tissue	2	9.5
	Other**	9	42.9

ECOG, Eastern Cooperative Oncology Group. Bajorin criteria includes: presence of visceral metastases and/or ECOG ≥ 2.

* Data shown are n of patients except for median and range values.

** One metastatic lesion in bladder, bone, mediastinum, pancreas, pelvis, peritoneum, kidney, suprarenal gland, and urethra was found.

**Table 2:** Prior anticancer therapy.

		No. of patients (n)*	%

**Systemic therapy**	Chemotherapy	21	100.0
	Chemotherapy/biological therapy	3	14.3

**Chemotherapy setting**	Advanced	16	76.2
	Adjuvant	4	19.0
	Neoadjuvant	1	4.8

**No. of agents of chemotherapy**	Median (range)	2 (1–4)	

**Agents administered to ≥ 10% of patients**	Cisplatin	17	81.0
	Gemcitabine	15	71.4
	Methotrexate	4	19.0
	Doxorubicin	3	14.3
	Carboplatin	3	14.3

**Surgery**	.	21	100.0

**Radiotherapy**	Palliative	5	23.8

* Data shown are n of patients except for median and range values.

**Table 3:** Adverse events regardless of relationship with plitidepsin observed in ≥ 5% of patients.

NCI-CTC	Patients (n = 21)									
	Grade 1		Grade 2		Grade 3		Grade 4		Total	

	n	%	n	%	n	%	n	%	n	%

**Abdominal pain**	3	14.3	2	9.5	1	4.8	.	.	6	28.6
**Alopecia**	2	9.5	2	9.5	NA	NA	NA	NA	4	19.0
**Anemia**	1	4.8	.	.	1	4.8	.	.	2	9.5
**Anorexia**	5	23.8	9	42.9	1	4.8	.	.	15	71.4
**Arthralgia**	4	19.0	3	14.3	.	.	.	.	7	33.3
**Constipation**	4	19.0	4	19.0	1	4.8	.	.	9	42.9
**Diarrhea**	2	9.5	2	9.5	1	4.8	.	.	5	23.8
**Dyspepsia**	2	9.5	2	9.5	.	.	.	.	4	19.0
**Dyspnea**	1	4.8	2	9.5	.	.	1	4.8	4	19.0
**Edema**	1	4.8	3	14.3	.	.	.	.	4	19.0
**Fatigue**	3	14.3	6	28.6	8	38.1	1	4.8	18	85.7
**Fever**	5	23.8	1	4.8	.	.	.	.	6	28.6
**General physical health deterioration**	.	.	1	4.8	4	19.0	1	4.8	6	28.6
**Hematuria**	8	38.1	.	.	.	.	.	.	8	38.1
**Muscle weakness**	1	4.8	2	9.5	1	4.8	.	.	4	19.0
**Myalgia**	4	19.0	1	4.8	1	4.8	.	.	6	28.6
**Nausea**	4	19.0	7	33.3	.	.	.	.	11	52.4
**Pain**	4	19.0	6	28.6	1	4.8	.	.	11	52.4
**Paresthesia**	6	28.6	4	19.0	.	.	.	.	10	47.6
**Proteinuria**	3	14.3	1	4.8	.	.	.	.	4	19.0
**Urinary tract infection**	3	14.3	1	4.8	1	4.8	.	.	5	23.8
**Vomiting**	3	14.3	4	19.0	1	4.8	.	.	8	38.1
**Weight decreased**	4	19.0	3	14.3	.	.	.	.	7	33.3

NCI-CTC, National Cancer Institute Common Toxicity Criteria.; NA, Not applicable.

**Table 4:** Biochemical and hematological abnormalities (per patient).

NCI-CTC	Patients (n = 21)									
	Grade 1		Grade 2		Grade 3		Grade 4		Total*	

	n	%	n	%	n	%	n	%	n	%

**Biochemical**										
**ALT increase**	9	42.9	3	14.3	1	4.8	.	.	13	61.9
**AP increase**	8	38.1	4	19.0	4	19.0	.	.	16	76.2
**AST increase**	7	33.3	4	19.0	.	.	.	.	11	52.4
**Bilirubin increase**	3	14.3	1	4.8	1	4.8	.	.	5	23.8
**CPK increase**	5	23.8	1	4.8	.	.	.	.	6	28.6
**Creatinine increase**	10	47.6	3	14.3	.	.	.	.	13	61.9

Hematological**										
**Anemia**	10	47.6	10	47.6	1	4.8	.	.	21	100.0
**Lymphopenia**	4	20.0	5	25.0	1	5.0	1	5.0	11	55.0
**Neutropenia**	1	5.0	.	.	.	.	.	.	1	5.0
**Thrombocytopenia**	4	19.0	.	.	.	.	.	.	4	19.0

* Total number of patients and cycles with data available for each parameter.

** No patients had leukopenia of any grade during the study. ALT, alanine aminotransferase; AP, alkaline phosphatase; AST, aspartate aminotransferase; CPK, creatine phosphokinase; NCI-CTC, National Cancer Institute Common Toxicity Criteria.
